# A lysine residue from an extracellular turret switches the ion preference in a Cav3 T-Type channel from calcium to sodium ions

**DOI:** 10.1016/j.jbc.2022.102621

**Published:** 2022-10-20

**Authors:** Wendy Guan, Kaidy G. Orellana, Robert F. Stephens, Boris S. Zhorov, J. David Spafford

**Affiliations:** 1 Department of Biology, University of Waterloo, Waterloo, Ontario, Canada; 2 Department of Biochemistry and Biomedical Sciences, McMaster University, Hamilton, Ontario, Canada; 3 Sechenov Institute of Evolutionary Physiology and Biochemistry, Russian Academy of Sciences, St. Petersburg, Russia; 4 Almazov National Medical Research Centre, St. Petersburg, Russia

**Keywords:** calcium channels, sodium channels, ion selectivity, AlphaFold2 molecular modeling, patch clamp, Cav, voltage-gated calcium channel, HEK, human embryonic kidney cell, Nav, voltage-gated sodium channel, PM, pore module, VSM, voltage-sensing module

## Abstract

Cav3 T-type calcium channels from great pond snail *Lymnaea stagnalis* have a selectivity-filter ring of five acidic residues, EE(D)DD. Splice variants with exons 12b or 12a spanning the extracellular loop between the outer helix IIS5 and membrane-descending pore helix IIP1 (IIS5-P1) in Domain II of the pore module possess calcium selectivity or dominant sodium permeability, respectively. Here, we use AlphaFold2 neural network software to predict that a lysine residue in exon 12a is salt-bridged to the aspartate residue immediately C terminal to the second-domain glutamate in the selectivity filter. Exon 12b has a similar folding but with an alanine residue in place of lysine in exon 12a. We express LCav3 channels with mutated exons Ala-12b-Lys and Lys-12a-Ala and demonstrate that they switch the ion preference to high sodium permeability and calcium selectivity, respectively. We propose that in the calcium-selective variants, a calcium ion chelated between Domain II selectivity-filter glutamate and aspartate is knocked-out by the incoming calcium ion in the process of calcium permeation, whereas sodium ions are repelled. The aspartate is neutralized by the lysine residue in the sodium-permeant variants, allowing for sodium permeation through the selectivity-filter ring of four negatively charged residues akin to the prokaryotic sodium channels with four glutamates in the selectivity filter. The evolutionary adaptation in invertebrate LCav3 channels highlight the involvement of a key, ubiquitous aspartate, “*a calcium beacon*” of sorts in the outer pore of Domain II, as determinative for the calcium ion preference over sodium ions through eukaryotic Cav1, Cav2, and Cav3 channels.

Eukaryotic voltage-gated calcium (Ca_v_) and sodium (Na_v_) channels belong to a large superfamily of P-loop channels, which play key roles in the physiology of electrically excitable cells (1, 2, 3). The pore-forming α-subunit of these ion channels folds from a single polypeptide chain of four homologous repeat domains DI, DII, DIII, and DIV. Each domain contains a voltage-sensing module (VSM) and contributes a quarter to the pore module (PM). A VSM has four transmembrane helices (S1-S4) connected by extracellular loops S1-S2 (L1), S3-S4 (L3), and intracellular loop S2-S3 (L2). The PM contains four transmembrane outer helices (S5), which are connected to four VSMs by linker-helices S4-S5 (L4), and four transmembrane inner helices (S6) whose cytoplasmic halves line the ion permeation pathway (the inner pore) and contribute to the bundle-crossing that occludes the ion channel pore in the closed state. Four membrane-reentering extracellular P-loops between helices S5 and S6 contain membrane-descending helices P1 linked to the S5 helix by loop S5-P1 (L5, also called turret) and membrane-ascending helix linked to helix S6 by loop P2-S6 (L6). Side chains of residues in short segments between the P1 and P2 helices contribute to the selectivity filter that along with other residues in the P-loops line the outer pore, which is open to the extracellular space. The distinct classes of calcium channels and sodium channels are defined by their selective passage of calcium and sodium ions (4) through the selectivity filters. In Na_v_ channels, the selectivity filter is composed of residues from the four domains: aspartate, glutamate, lysine, and alanine, *i.e.*, the DEKA ring (5). In Ca_V_ channels, the selectivity-filter ring has five acidic residues, *e.g.*, EE(D)EE or EE(D)DD (6).

Many animal groups such as nematodes, parasitic platyhelminth (*e.g.*, schistosomes), hemichordates, and echinoderms completely lack voltage-gated Na_V_2 or Na_V_1 channel genes in their genomes (7). It is well established that nematodes generate cardiac-like action potentials in pharyngeal muscles that require a voltage-dependent Na^+^ current, but the gene responsible for this Na^+^ current has eluded discovery (8, 9). Similarly, the giant pond snail, *Lymnaea stagnalis*, possesses cardiac action potentials requiring Na^+^ and Ca^2+^, but its only Na_V_1 sodium channel gene in the nervous system does not express in the cardiovascular system (10). We have demonstrated that the source of the Na^+^ current in the snail heart is a low voltage-activated Cav3 T-type channel that can be blocked by nickel or drugs, *e.g.*, mibefradil (10). The T-type currents in cardiomyocytes possess characteristic biophysical properties that enable their participation in rhythm generation to serve a pacemaking role for the molluscan heart (10).

Measurement by quantitative PCR reveals that the only isoform of the Ca_V_3 T-type channel that is expressed in the snail heart has an alternatively spliced isoform of the LCav3 T-type channel gene containing exon 12a (10). The high Na^+^ permeation of T-type currents from the snail heart can be achieved *in vitro* by expressing the snail LCav3 channel harboring exon 12a. Exon 12b, which does not express in the snail heart, but predominates in skeletal muscle tissue, exhibits the typical phenotype of a mostly Ca^2+^ currents in the LCav3 T-type channel (10). We demonstrate that the human calcium-selective Cav3.2 channel can be converted into channel with a preference for passage of Na^+^ over Ca^2+^, in chimeras, which include exon 12a from the snail LCa_V_3 channel (11). Exons 12a and 12b code for the L5 extracellular turret in domain II (IIS5-P1) of the Cav3 T-type channel, terminating just upstream of the selectivity filter residues (10, 11). What has eluded discovery is an atomic model to explain how a typically fast, Ca^2+^-selective T-type channel converts into a kinetically slow ion channel with a preference for passage of Na^+^ over Ca^2+^ without alterations to the selectivity filter or in any Voltage-Sensor Domain (VSD).

Here, we employ multiple sequence alignments, 3D molecular modeling with the AlphaFold2 neural network software, and mutational analysis of expressed Cav3 channels to show that what varies is the presence of a single lysine residue within exon 12a, but not exon 12b to neutralize a critical aspartate residue in the ED motif of Domain II contributing to calcium selectivity. Appearance of short, alternative extracellular loops evolved to infiltrate and adjust the pore’s ion permeability, provides an ingenious and economical way for nematodes and mollusks to generate multiple phenotypes resembling various Ca_V_ and Na_V_ channels within their only T-type channel in the absence of expression of differing Ca_V_ or Na_V_ channel isoforms.

## Results

### Conservation of asymmetrical pattern of extracellular loops in eukaryotic Ca_V_ and Na_V_ channels

**Figure 1:**
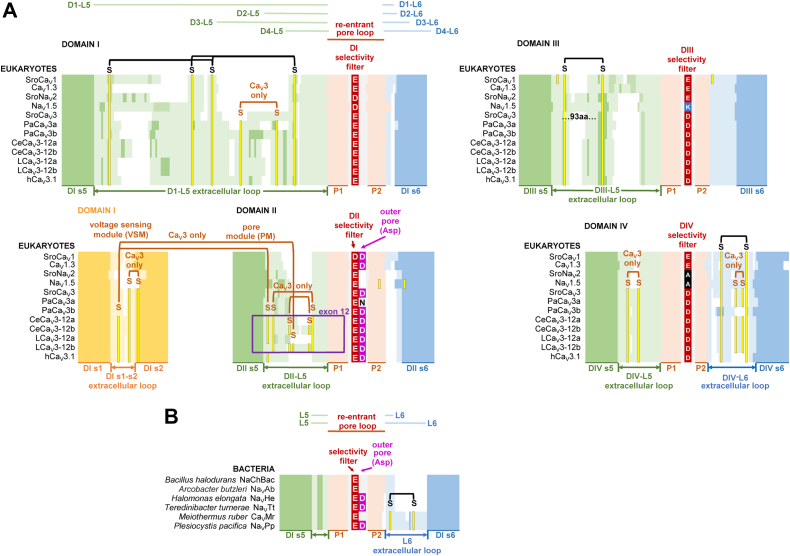
**Aligned sequences ofeukaryotic andbacterial Ca**_**V**_**and Na**_**V**_**channels.** Multiple sequence alignment illustrates the consistency in the relative sizes of extracellular loops, number of cysteine bridges, and pore selectivity filter residues between the (*A*) four domains of the most phylogenetically distant eukaryotes (choanoflagellate: SroCa_V_1, SroCa_V_3, and SroNa_V_2) and human homologs (Ca_V_1.3, Na_V_1.5, and Ca_V_3.1). It also illustrates variations in invertebrate Ca_V_3 homologs, such as anthozoan cnidarians (PaCa_V_3a/3b, *Porites australiensis*), nematodes (CeCa_V_3-12a/12b, *Caenorhabditis elegans*), and mollusks (LCa_V_3-12a/12b, *Lymnaea stagnalis*) compared to (*B*) the single pore domain of bacterial Ca_V_ and Na_V_ channels. Each pore domain is flanked by outer, S5 (*dark green color*), and inner, S6 (*dark blue color*), membrane spanning alpha helices. L5 extracellular loops (*light green color*) extend from the outer helix (S5) to pore helix 1 (P1, *salmon color*) and L6 extracellular loops (*light blue color*) extend from pore helix 2 (P2, *salmon color*) to the inner helix (S6). The downward angled pore helices (*salmon color*) converge on a constricted access pathway for ion passage, containing pore-lining selectivity filter residues consisting mostly of side chains of negatively charged glutamates (E, *red color*) or aspartates (D, *red color*), with a characteristic divergence in the selectivity filter of eukaryotic Na_V_ channels with a positively charged lysine (K, *blue color*) in Domain II or III and a neutral amino acid (*e.g.,* alanine, A, *black color*) in Domain IV replacing negatively charged residues of calcium channels. The +1 position of the selectivity filter of Domain II is always a negatively charged aspartate (D, *magenta color*) residue in the outer pore of all eukaryotic calcium channels (Ca_V_1, Ca_V_2, and Ca_V_3), with a notable exception being one of two Ca_V_3 channel genes in anthozoans (cnidarians), where the negatively charged aspartate residue (D) in the outer pore is replaced by a more neutral asparagine residue (N). Cysteines (*yellow*) contribute to a conserved set of four intraturret bridge pairs located within known eukaryotic Na_V_ and Ca_V_ channels, and these are mostly contained in the longest of the two L5 extracellular loops (DI, DIII) facing across the pore domain from each other. All known Ca_V_3 channels possess from two to six extra cysteine bridges above this core set of four cysteine bridges. All but one of these novel Ca_V_3 channel- specific cysteine pairs is concentrated in the shortest of the extracellular loop pairs (DII, DIV), also lying across from each other in the pore domain. One of these novel cysteine pairs in most Ca_V_3 channels bridges an extracellular loop of the voltage sensor domain (IS1-S2) and the DII L5 extracellular loop of the pore domain. It is in this shortest of the L5 extracellular loops in Domain II, where a positively charged lysine contained in alternatively spliced exon 12a lies in proximity to neutralize the outer pore aspartate residue to generate sodium permeable Ca_V_3 T-type channels.

Cryo-EM structures reveal a common, asymmetrical pattern of extracellular loops forming a canopy that partially encloses the selectivity filters below, contributed by the four homologous domains of eukaryotic Na_V_1 (12), Ca_V_1 (13), Ca_V_2 (14) and Ca_V_3 (15) channels. The structural conservation of extracellular loops extends outside the animals to the basal, single-cell choanoflagellates, such as *Salpingoeca rosetta*, which possess animal homologs, SroCa_V_1, SroCav3, and SroNav2, as illustrated in the multiple alignment in Fig. 1. The four pore domains within different Ca_V_ and Na_V_ channels in eukaryotes (Fig. 1*A*) significantly vary from those in bacteria (Fig. 1*B*) by the presence of long L5 extracellular loops (light green, Fig. 1). The longest of the L5 extracellular loops in eukaryotes is within Domain I (L5_I_) followed by Domain III (L5_III_), and these are stabilized by a conserved set of two and one disulfide bonds, respectively, in known eukaryotic Na_V_ and Ca_V_ channel homologs (black color, Fig. 1*A*). The longest of the L6 extracellular loops in eukaryotes is in Domain IV (L6_IV_), which also is stabilized by a disulfide bond common within eukaryotic Ca_V_ and Na_V_ channels (Fig. 1*A*). Bacterial homologs do not possess extensive L5 extracellular loops like the animals but have a similar disulfide bond (black color, Fig. 1) in the L6 extracellular loop of extended length in some (16) but lacking in other (17, 18) bacterial homologs (Fig. 1*B*).

### Unique, additional disulfide bonds within the shorter extracellular loops of Domains II and IV in Ca_V_3 channels

Known Ca_V_3 channels contain two to six additional disulfide bonds (dark orange color, Fig. 1*A*) above the core of four disulfide bonds (black color, Fig. 1*A*) in extracellular loops shared with other eukaryotic Ca_V_ and Na_V_ channels. These unique “*Cav3 only*” disulfide bonds reside, in all but one case, in the shortest of the extracellular loops of Domain II (L5_II_) and Domain IV (L5_IV_ and L6_IV_), which lie across from each other in the pore domain, and at a lower profile, closer to the membrane and pore selectivity filter than the larger L5_I_ and L5_III_ extracellular loops (Fig. 1*A*). Cryo-EM structures of human Cav3.1 (15) and Cav3.3 (19) channels reveal one of these unique cysteines in the short L5_II_ extracellular loop to form a disulfide bond with a cysteine (yellow color, Fig. 1*A*) in the extracellular loop S1-S2 of VSM-I (dark yellow color, Fig. 1*A*). This unique tethering of the voltage-sensor and PMs likely contributes to the reported redox sensitivity and variability in ion channel kinetics of Ca_V_3 T-type channels (20, 21).

### Exon 12 splicing can modify the ion preference of molluscan and nematode Ca_V_3 channels

Exons 12a and exons 12b specifically vary in one residue within all molluscan (*e.g.*, *L. stagnalis*) (10) and nematode (*e.g.*, *Caenorhabditis elegans*) (22) Cav3 channels. This is a lysine residue within exon 12a (K1067, Genbank Acc. # JX292155 and K819, Genbank Acc. # AAP84337, respectively) compared to a neutral alanine or methionine residue at a similar position within exon 12b (A1078, Genbank Acc. # AAO83843 or M826, Genbank Acc. # AAP79881, respectively).

### AlphaFold2 models of Cav3 splice variants from L. stagnalis and C. elegans

**Figure 2:**
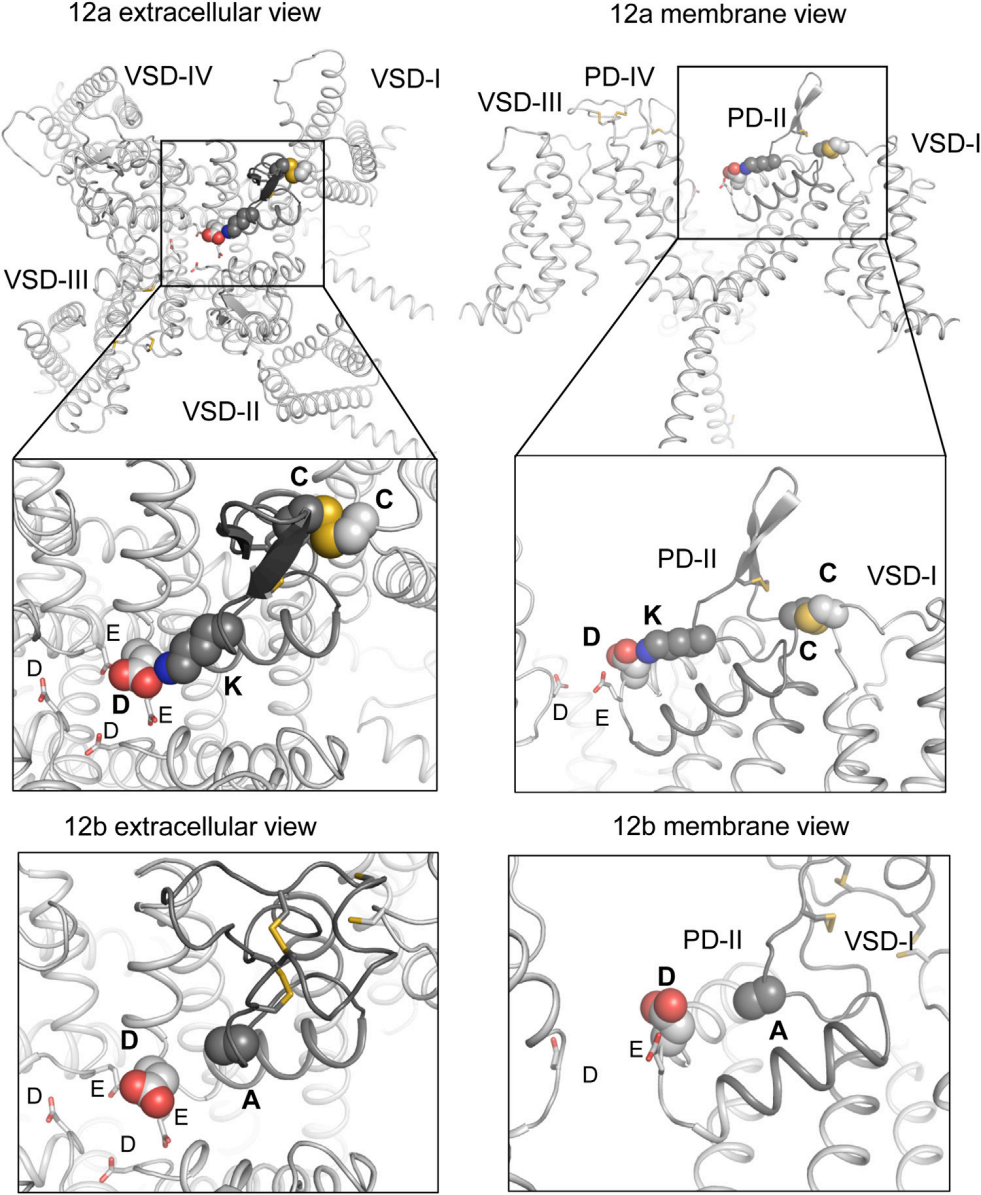
**AlphaFold2 models of molluscan LCa**_**V**_**3 channel with exon 12a or exon 12b.** Sodium-permeable channel with exon 12a (*dark*) has a lysine salt-bridged to the aspartate, which is immediately C terminal to the second-domain glutamate in the EE(D)DD selectivity-filter ring. In the calcium-selective channel with exon 12b (*dark*), an alanine is at the place of lysine in exon 12a. In exon 12a, a cysteine is disulfide-bonded with VSM-I. See text for more details. VSM, voltage-sensing module.

Figure 2 shows models of calcium-selective and sodium-permeable variants of Cav3 channel from *L. stagnalis* with exons 12a and 12b, respectively. Both exons are located in the extracellular loop L5_II_ that descends from the extracellular space to the groove between helices IIP1 and IIP2. In the channel with exon 12a, the long side chain of the lysine residue at the apex of loop L5_II_ is salt bridged to the second-domain aspartate of the selectivity-filter ring EE(D)DD and neutralizes the negative charge at this aspartate, a fingerprint residue of eukaryotic calcium channels. In the channel with exon 12b, loop L5_II_ has a similar folding, but alanine, which is located at position of lysine in exon 12a (Fig. 2), is far from the second-domain aspartate and does not affect the negative charge at the aspartate.

AlphaFold2 models of nematode Cav3 channels like *C. elegans* with exons 12a and 12b predicted folding that overlaps with that of the molluscan LCav3 channels. In the Ca_V_3 channels from both phylogenetic groups, a lysine from exon 12a is salt-bridged to the second-domain aspartate in the selectivity filter. In the Cav3 channel from *C. elegans*, a methionine in exon 12b is engaged in a hydrophobic contact with valine one turn of helix IIP2 downstream from the aspartate and does not neutralize its negative charge.

Despite differing sequences and number of intraturret cysteine bridges, the AlphaFold2 neural network predicted identical positioning of the neutral amino acid in exon 12b and the charged lysine in exon 12a whose ammonium group is ∼ 2.6 Å from a carboxylate oxygen in the aspartate residue found in similar position of the outer pore in cryo-EM structures of calcium channels Ca_V_1 (13), Ca_V_2 (14) and Ca_V_3 (15). Thus, the AlphaFold2 models suggest that it is the salt-bridge between the lysine in exon 12a and the second-domain aspartate in the selectivity filter ring EE(D)DD that renders sodium permeability to T-type channel isoforms with exon 12a.

### Alternative-splicing in exon 12 changes kinetic properties in invertebrate Ca_V_3 channels

**Figure 3:**
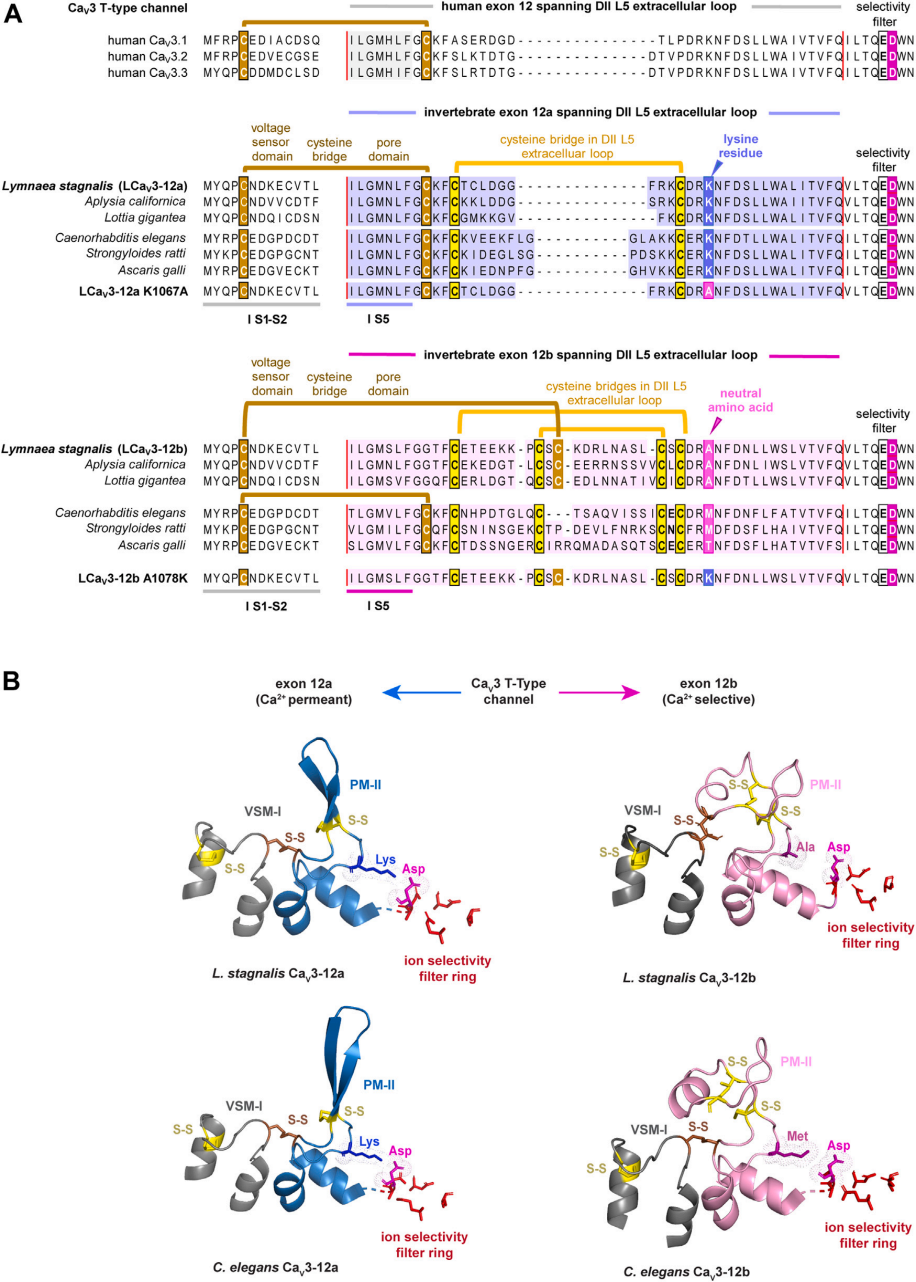
**Alternative exon 12a and exon 12b coding for alternative L5 extracellular loops of molluscan and nematode Cav3 T-type channels.***A*, aligned amino acid sequences for human (Cav3.1, Cav3.2, and Cav3.3) and singleton homologs of Cav3 T-type channels from representative mollusks (*Lymnaea stagnalis*, *Aplysia californica,* and *Lottia gigantea*) and nematodes (*Caenorhabditis elegans*, *Strongyloides ratti,* and *Ascaris galli*). Borders for exon 12 indicated by *red lines*. *B*, positively charged lysine residue in exon 12a (*blue color*) but not the neutral amino acid (A, M, T) in exon 12b (*magenta color*) lies adjacent to the outer pore aspartate residue in AlphaFold2 structural models where the negative side chains are positioned to neutralize the outer pore aspartate residue and create sodium permeant Cav3 T-type channels. Locations of a cysteine bridge (*brown color*) spanning the voltage sensor domain and the pore domain are indicated for the molluscan exon 12a isoform, nematode exon 12b isoform, and human Ca_V_3 channels.

Protostome invertebrates from flatworms, nematodes, arthropods, and mollusks to annelids contain alternative L5_II_ loops encoded by exons 12a or 12b (10, 11). In the AlphaFold2 models, exon 12a in molluscan (*e.g.*, *L. stagnalis*) and nematode (*e.g.*, *C. elegans*) Ca_V_3 channels possess a similarly placed cysteine disulfide-bonded with a cysteine in the S1-S2 extracellular loop of VSM-I like in mammalian Ca_V_3 channels (15) (Fig. 3*A*). The disulfide bonding of VSM-I to exon 12b is contained within a pair of intraturret cysteine bridges in most protostome invertebrates such as mollusks (*e.g.*, *L. stagnalis*) rather than outside the intraturret cysteine bridge of exon 12a. The exception is in nematode Cav3 channels (*e.g.*, *C. elegans*) in which the cysteine disulfide-bonded to VSM-I is in the same position for exon 12a and exon 12b outside the one or two pairs of intra-L5_II_ loop cysteine bridges, respectively (10, 11) (Fig. 3). We previously reported that all measured kinetic features (activation, inactivation, deactivation, and recovery from inactivation) to a significant degree are slowed in molluscan LCa_V_3 channels with exon 12a compared to the faster kinetics of the channels with exon 12b, in which the cysteine bridging to the VSM-I is localized within the double set of intraturret cysteines.

### Estimations of the relative change in sodium ion permeation by changes in response to external Na^+^ replacing NMDG^+^

**Figure 4:**
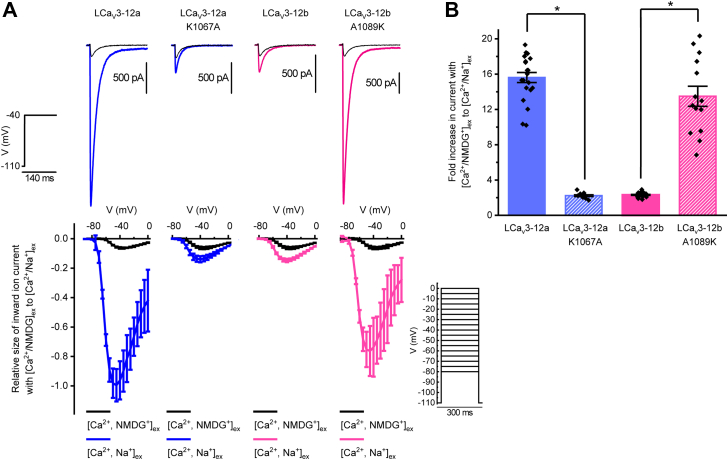
**Mutational analyses illustrating the intraconvertibility of exon 12a and exon 12b within molluscan LCav3 channels in generating highly sodium-permeant Cav3 T-type channels with a lysine residue in homologous position within the DII L5 extracellular loop.** The relative degree of Na^+^ *versus* Ca^2+^ ion permeation was estimated in the fold change in peak current size (elicited from a voltage step to −40 mV from a holding potential of −110 mV) in the presence of external Na^+^ ions (Ca^2+^ and Na^+^ containing external solution) *versus* when large and weakly permeant monovalent ion, NMDG^+^, replaces external Na^+^ ions (Ca^2+^ and NMDG^+^ containing external solution). *A*, representative ionic current traces of WT and mutagenized LCa_V_3 channels and their current–voltage relations of their relative current size ensembles ±SEM. *Black-colored* current traces were recorded in the presence of external Ca^2+^ and weakly permeant cation (NMDG^+^), and the *blue-colored* (exon 12a containing) and *magenta-colored* (exon 12b containing) traces were recorded after replacement of NMDG^+^ with equimolar Na^+^. *B*, bar graphs ± SEM with scatterplot of replicates of recorded cells. Statistical significance was evaluated in a parametric one-way ANOVA with Tukey test, *F*(3,64) = 152.7, *p* < .0000001. Statistically significant differences (*P* < 0.05) are illustrated by (∗).

To confirm the proposed role of lysine residue in rendering sodium permeability to the channels with exon 12a, we generated and expressed in the human embryonic kidney (HEK)-293T cells mutant channels LCa_V_3-12a_K1067A and LCa_V_3-12b_A1078K in which the lysine residue of exon 12a and alanine residue in exon 12b were swapped. The relative sodium and calcium permeation through LCa_V_3 channels was estimated as the relative fold increase in peak current amplitude when external Na^+^ ion replaced weakly permeant cation, NMDG^+^ (N-methyl-D-glucamine) in the presence of external Ca^2+^. Representative peak current traces (Fig. 4*A*) and current-voltage relationships (Fig. 4*B*) illustrate a significant fold decrease in peak current size in presence of external Na^+^ ions when lysine is replaced by alanine in the LCav3-12a_K1067A mutant, (2.24 ± 0.10, n = 9) compared to wildtype LCa_V_3-12a (15.62 ± 0.57, n = 21). LCav3-12a_K1067A mutant did not significantly vary from the native LCa_V_3-12b (2.32 ± 0.03, n = 26), suggesting that the only structural feature that is responsible for the changed Na^+^ to Ca^2+^ ion preference between exon 12a and exon 12b splice isoforms is the loss of the lysine residue in the L5_II_ loop. And similarly, exchanging the lysine in position of the alanine within the exon 12b in the LCa_V_3-12b_A1089K mutant yielded sodium permeant T-type channels (13.49 ± 1.14, n = 14). The latter had a fold increase in peak current amplitude in the presence of external Na^+^ that was dramatically different from the wildtype LCa_V_3-12b channel (2.32 ± 0.054, n = 22) but not significantly different from native LCa_V_3-12a (15.66 ± 0.43, n = 21).

### Ca^+^ to monovalent ion (X^+^) permeabilities quantified using bi-ionic reversal potentials

**Figure 5:**
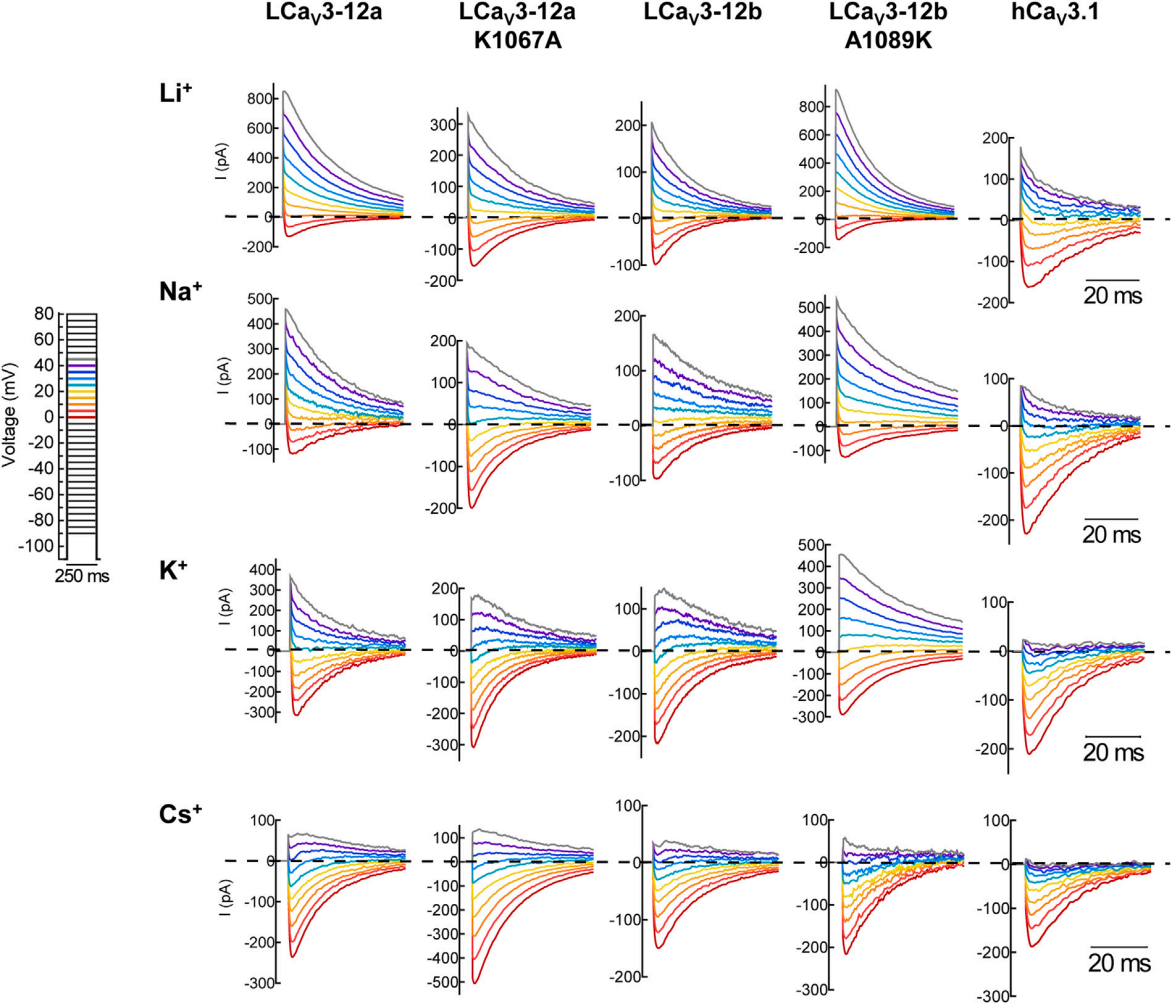
**Illustration of representative ensembles of ionic currents generated in different voltage steps that cross at or near the reversal potential in bi-ionic recording conditions used to estimate the relative permeability change of divalent cation (Ca**^**2+**^**) influx to monovalent ion (Li**^**+**^**, Na**^**+**^**, K**^**+**^**, and Cs**^**+**^**) efflux in Ca**_**V**_**3 channels.** Ionic currents of transfected Cav3 channels in HEK-293T cells were recorded using whole cell voltage clamp, in bi-ionic recording solutions, where high Ca^2+^ in external solutions (4 mM) and a high concentration (100 mM) of monovalent ions (Li^+^, Na^+^, K^+^, and Cs^+^) in internal solutions generates a reversal potential elicited from a series of current-generating voltage steps that is considered to reflect the relative permeability of Ca^2+^ influx normalized to the permeability for the monovalent ion efflux (23).

A means to estimate an ion channel permeability to divalent ions (Ca^2+^), compared to monovalent ions (X^+^), P_Ca_/P_X_, relies on a measurement of the reversal potential in bi-ionic recording conditions (23). High Ca^2+^ in external solutions (4 mM) and a high concentration (100 mM) of monovalent ions (Li^+^, Na^+^, K^+^, Cs^+^) in internal solutions generate a reversal potential elicited from a series of current-generating voltage steps that is considered to reflect the relative permeability of Ca^2+^ influx normalized to the permeability for the monovalent ion efflux (23). Notably, Cs^+^ was observed as a weakly permeable ion through all Cav3 channels (illustrated by the predominance of inward Ca^2+^ current elicited by voltage steps near the reversal potential in the bottom row of current traces in Fig. 5). Human Cav3.1 channels are not very permeable to any of the monovalent ions tested above (illustrated by the predominance of inward Ca^2+^ currents elicited in voltage steps generated near the reversal potential in the column of current traces on the right side in Fig. 5).

In contrast, monovalent ions (Li^+^, Na^+^, K^+^) dramatically influence the reversal potential of molluscan LCav3 channels with the lysine residue in the L5_II_ extracellular loop (LCav3-12a and LCav3-12b_A1089K). These channels demonstrate statistically significantly higher monovalent ion permeability for Li^+^, Na^+^, and K^+^ than the LCav3 channels with alanine in similar position of the L5_II_ loop (LCav3-12b and LCav3-12a_K1067A). As illustrated in Figure 4, the markedly higher monovalent ion permeability of LCav3 channels with lysine in the L5_II_ loop (LCav3-12a and LCav3-12b_A1089K) is reflected in the large outward monovalent ion conductances observed for Li^+^, Na^+^, and K^+^ predominating over inward Ca^2+^ conductances elicited in voltage steps generated near the reversal potential. The higher monovalent ion permeabilities of LCav3-12a and LCav3-12b A1089K channels are also reflected in the observed steeper slope of the outward whole cell conductances of Li^+^, Na^+^, and K^+^ ions above the reversal potential (see full-scale current voltage relationships, Fig. 5), and the statistically significant leftward, hyperpolarizing shift in reversal potential (close-up view near the reversal potential, Fig. 5).

**Figure 6:**
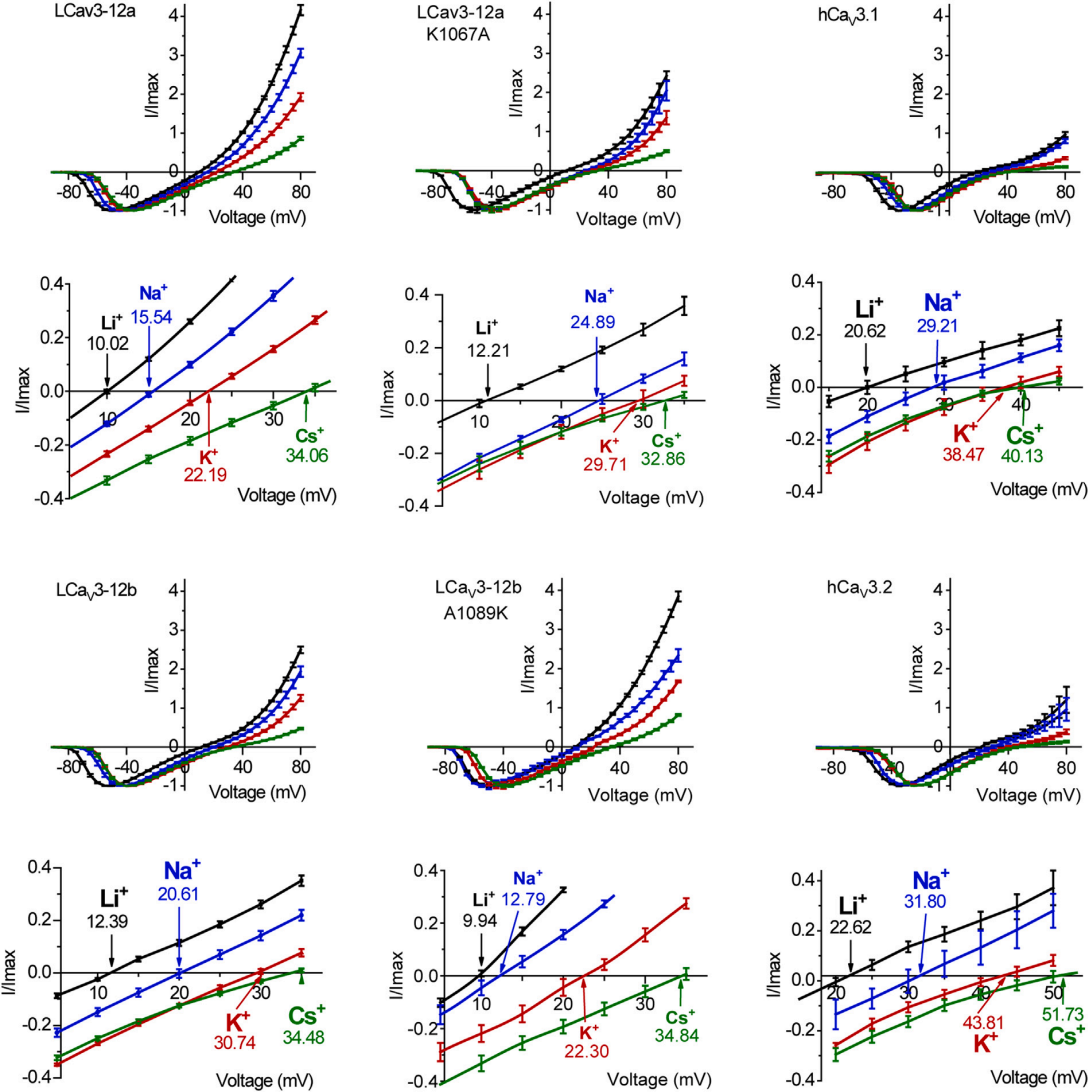
**Current–voltage relationships illustrated in a full-scale (*above*) and closeup view (*below*) of the relative peak current sizes of Ca**_**V**_**3 channels elicited near the reversal potentials.** Mean values illustrated by *arrows* in closeup view. Ensembles of ionic currents were generated in a series of voltage steps that cross near the reversal potential in bi-ionic conditions illustrate the relative permeability change of divalent cation (Ca^2+^) influx to monovalent ion (Li^+^, Na^+^, K^+^, or Cs^+^) efflux in wildtype and mutagenized LCav3-12a/12b as well as human Ca_V_3.1 and Ca_V_3.2 channels.

The position of reversal potentials (sampled in representative current traces, Figure 4, extrapolated from current-voltage relationships Figure 5, and summarized in bar graph form, Fig. 6 inset) are considered a reflection of the relative contribution of inward divalent cation conductance (Ca^2+^) compared to the outward monovalent ion conductances (Li^+^/Na^+^/K^+^/Cs^+^) and used to calculate the relative permeabilities of inward Ca^2+^ flux to monovalent ion (X^+^) flux (P_Ca_/P_X_) (illustrated as bar graph ± s.e.m. overlaid with scatter plot of replicates, Fig. 6) according to a bi-ionic equation provided by Fatt and Ginsborg (1958) (23). The single lysine residue in the extracellular loop of wildtype LCav3-12a and LCav3-12b A1089K mutant compared to the alanine residue in similar position of wildtype LCav3-12b and LCav3-12a K1067A mutant generate statistically significant increases in the relative permeabilities for monovalent ions, Li^+^, Na^+^, and K^+^, over the relative Ca^2+^ permeability. Reversal potentials and calculated relative permeability ratios (PCa^2+^/PX^+^) are also provided for Ca_V_3.1 and Ca_V_3.2 channels in Figure 6, illustrating the much lower relative permeabilities measured for monovalent ions, especially Li^+^/Na^+^/K^+^ in the highly calcium-selective, human homologs than their invertebrate counterparts, measured using the bi-ionic reversal potential method.

### The lysine residue in the L5_II_ extracellular loop of exon 12a generates more sodium permeant LCa_V_3 channels by neutralizing the aspartate residue in the Domain II ED motif of the selectivity filter

**Figure 7:**
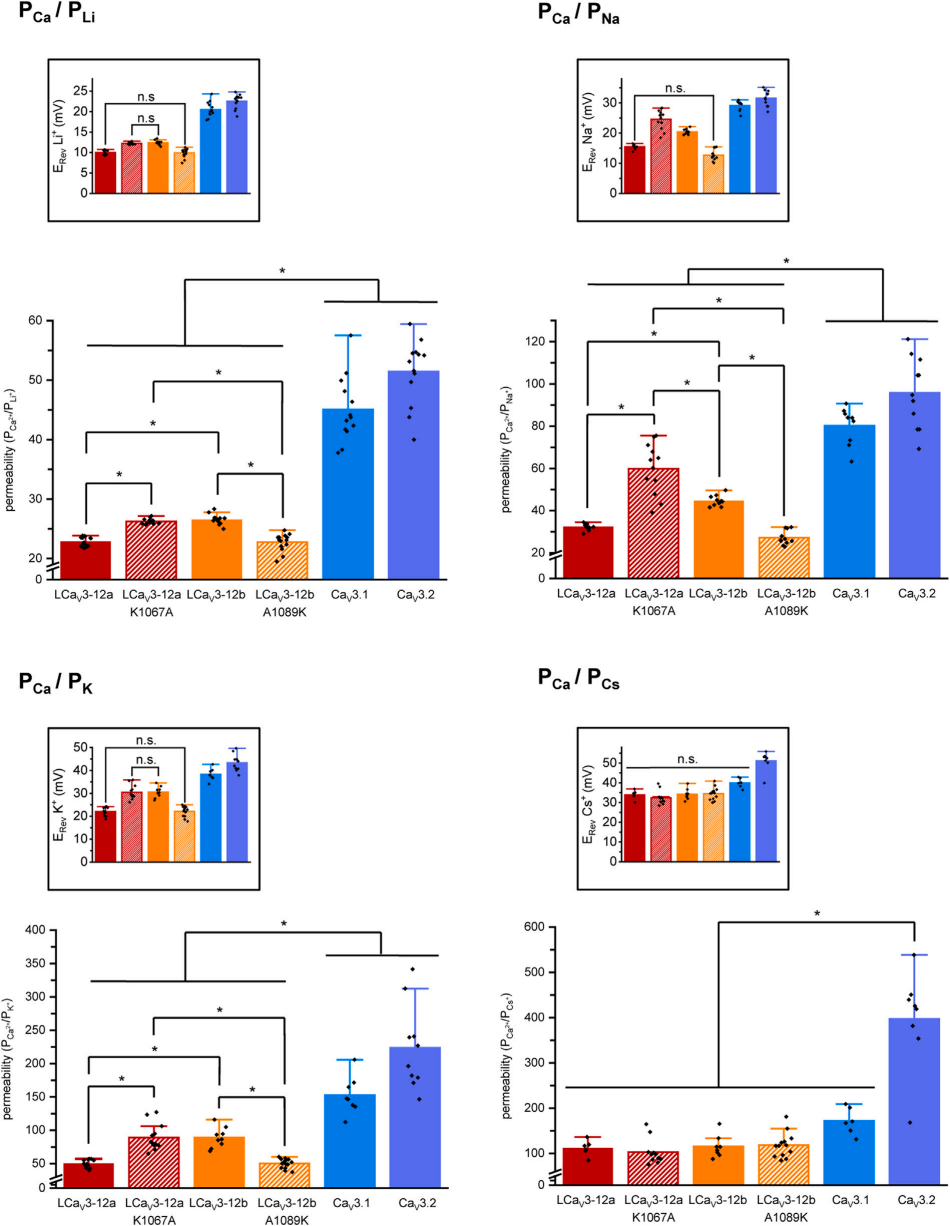
**Calculated relative divalent ion (Ca**^**2+**^**) to monovalent ion (Li**^**+**^**, Na**^**+**^**, K**^**+**^**, and Cs**^**+**^**) permeabilities derived from reversal potentials (inset) extrapolated from current voltage relationships (illustrated in**Fig. 6**) of wildtype and mutagenized molluscan LCav3-12a/12b channels and human homologs (Cav3.1, Cav3.2).** The single lysine residue in the extracellular loop of wildtype LCav3-12a and LCav3-12b A1089K mutant compared to the alanine residue in similar position of wildtype LCav3-12b and LCav3-12a K1067A mutant generates statistically significant increases in the relative permeabilities for monovalent ions, Li^+^, Na^+^, and K^+^, compared to the permeability of Ca^2+^. Statistical significance was evaluated in a parametric one-way ANOVA with Tukey test: for Li^+^: F(5,76) = 204.6, *p*-value < 0.0000001; for Na^+^: F(5,58) = 86.3, *p*-value < 0.0000001; for K^+^: F(5,61) = 61.6, *p*-value < 0.0000001; for Cs^+^: F(5,46) = 46.2, *p*-value < 0.0000001. The illustrated Erev data (insets) that did not meet statistical significance (*p* < 0.05) are labeled as n.s. or not significant. Statistically significant differences (*p* < 0.05) are illustrated by (∗).

Our experiments confirm the molecular-modeling prediction that swapping the alanine with the lysine of exon 12a onto exon 12b generates a LCa_V_3 channel with a preference for passage of Na^+^ over Ca^2+^, reminiscent of LCav3-12a, while the reverse (lysine to alanine) mutation in the extracellular L5_II_ loop generates a more traditional Ca^2+^-selective Cav3 channel onto LCav3-12a, reminiscent of LCav3-12b. Despite the divergence in their sequences and differing one and two intraturret cysteine bridges in exon 12a and exon 12b, respectively, the L5_II_ extracellular loop from both exons have similar backbone folding so that the C^α^ atom of lysine in exon 12a is in identical position of alanine C^α^ atom of exon 12b (Fig. 7).

**Figure 8:**
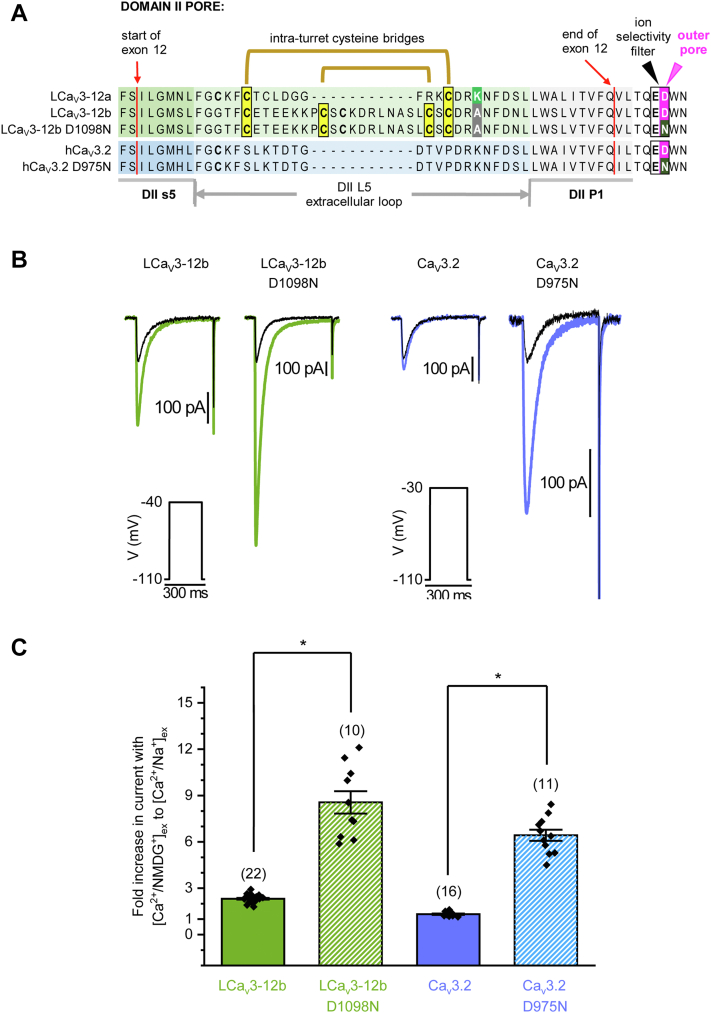
**Neutralizationof a negative charge in the outer pore, aspartate (D) to asparagine (N) substitution in the ion selectivity filter +1 position of domain II, dramatically increases the peak ionic currents of molluscan LCa_V_3-12b and human Ca_V_3.2 channels in the presence of sodium ions replacing weakly permeant monovalent ion, NMDG^+^ in Ca^2+^ ion containing external solutions.** The relative degree of Na^+^ *versus* Ca^2+^ ion permeation can be estimated as illustrated in the fold change in peak current size (elicited from a voltage step to −40 mV (LCa_V_3) or −30 mV (hCa_V_3.2) from a holding potential of −110 mV) in the presence of external Na^+^ ions (Ca^2+^ and Na^+^ containing external solution) *versus* when large and weakly permeant monovalent ion, NMDG^+^, replaces external Na^+^ ions (Ca^2+^ and NMDG^+^ containing external solution). *A*, amino acid alignments of WT and mutagenized LCa_V_3 and Ca_V_3.2 channels spanning exon 12 and the pore selectivity filter in domain II. *B*, representative ionic current traces of WT and mutagenized LCa_V_3 and Ca_V_3.2 channels and (*C*) bar graph of mean ± SEM overlayed with a scatterplot of the data. Statistical significance was evaluated in a parametric one-way ANOVA with Tukey test, *F*(3,50) = 108.9, *p* < 0.0000001. Statistically significant differences (*P* < 0.05) are illustrated by (∗). *Black-colored* current traces in (*B*) were recorded in the presence of external Ca^2+^ and weakly permeant cation (NMDG^+^), and the *blue-colored* (exon 12a containing) and *magenta-colored* (exon B containing) traces were recorded after replacement of NMDG^+^ with equimolar Na^+^.

To mimic the consequence of the charge neutralization of the outer pore aspartate by the lysine residue in the L5_II_ extracellular loop of exon 12a, we further mutated the second-domain aspartate in the selectivity filter to asparagine in the calcium-selective T-type channel isoform in mollusk (LCav3-12b_ D1098N) and human (Cav3.2_D975N), see Figure 8*A*. A similar and dramatic increase in peak current size was observed in the presence of external Na^+^ ions (Ca^2+^ and Na^+^ containing external solution) *versus* the case when large and weakly permeant monovalent ion NMDG^+^ replaced external Na^+^ ions (Ca^2+^ and NMDG^+^ containing external solution) in mutants where the ED aspartate is replaced by asparagine (EN) in the ion selectivity filter of both molluscan LCav3 and human Cav3.2 channels (Fig. 8). The fold increase in peak current in presence of external Na^+^ ions was significantly higher for LCa_V_3-12b D1098N mutant (8.56 ± 0.73, n = 17) compared to wildtype molluscan LCa_V_3-12b (2.32 ± 0.054, n = 22) (Fig. 8). The fold increase in peak current in presence of external Na^+^ ions was similarly and significantly higher for hCa_V_3.2 D975N mutant (6.53 ± 0.362, n = 16) than wildtype human Ca_V_3.2 (1.32 ± 0.044, n = 16) (Fig. 8).

## Discussion

### A short IIS5-P1 loop evolved to co-opt a Cav3 T-type channels into a slow Na_V_ channel phenotype without altering the pore’s selectivity filter

We demonstrate here a first example of how nature has engineered a means for an ion channel to switch its phenotype with the positioning of a single lysine residue in an alternative exon to enable the dominance of passage of Na^+^ over Ca^2+^, utilizing a short alternative extracellular L5 loop in Domain II.

AlphaFold2 modeling predicts that swapping the alanine with lysine from exon 12a to exon 12b generates a LCa_V_3 channel with a preference for passage of Na^+^ over Ca^2+^, reminiscent of LCav3-12a, while the reverse (lysine to alanine) mutation in the extracellular L5_II_ loop generates a more traditional Ca^2+^-selective Cav3 channel onto LCav3-12a, reminiscent of LCav3-12b.

**Figure 9:**
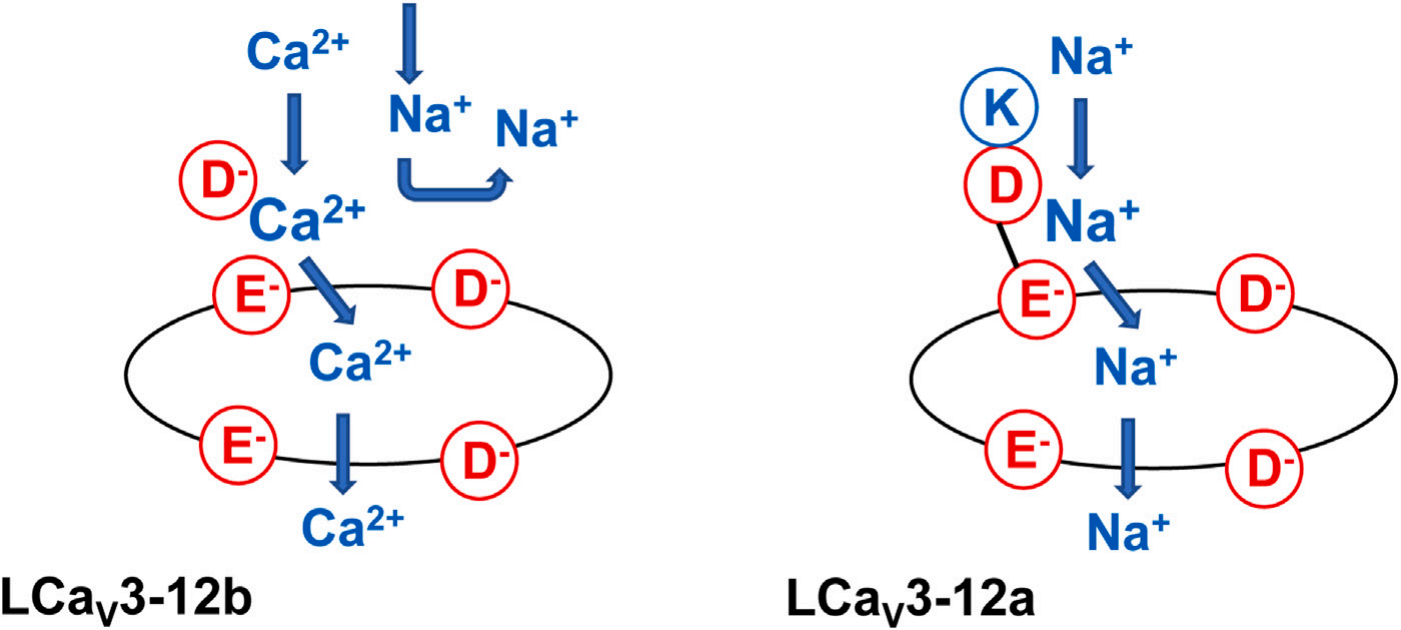
**Possible mechanism for the different ion selectivity phenotypes of molluscan LCav3 channels with exons 12b and 12a.** Ca^2+^ ions are chelated between carboxylate groups in the dipeptide ED of Domain II in LCa_V_3-12b. Incoming Ca^2+^ ions knock out the chelated Ca^2+^ ions, which move down into the inner pore of the channel. Incoming Na^+^ ions are repelled by the chelated Ca^2+^ ions. In the LCa_V_3 channel with exon 12a, the lysine residue from the exon is salt bridged to the ED aspartate and neutralizes its negative charge. The ring of four acidic residues would be sodium permeable like the ring of EEEE residues in the selectivity filter of prokaryotic sodium channels.

The atomic models suggest the following mechanisms for the switch in preference for passing Ca^2+^ or Na^+^ in the Cav3 channels with exons 12a and 12b (Fig. 9). In LCav3 with exon 12a, Ca^2+^ is chelated in the selectivity filter between carboxylate groups in the dipeptide ED of the Domain. An incoming Ca^2+^ knocks out the chelated Ca^2+^, which moves down into the inner pore, while an incoming Na^+^ is repelled by the chelated Ca^2+^. In LCav3 with exon 12b, the lysine residue from the exon is salt bridged to the ED and neutralizes its negative charge. The selectivity-filter ring with the net negative charge of −4 would be sodium-permeable like prokaryotic sodium channels with the EEEE residues in the selectivity-filter ring (24).

We illustrate that partial neutralization of the aspartate (D) by site-specific mutation to an asparagine (N) in the outer pore increases the preference for passage of Na^+^ over Ca^2+^ in a manner consistent with the modeling prediction of the charge neutralization of the aspartate by the juxtaposition of the positively charged lysine residue in the extracellular L5_II_ loop within exon 12a of the molluscan Cav3 T-type channel.

### Physiological experiments demonstrate a change in ion selectivity from Ca to Na channels due to neutralization of the Domain II selectivity-filter aspartate with the turret lysine

The charge neutralization of the selectivity-filter aspartate in Domain II by the juxtaposed lysine instead of alanine of the extracellular turret within exon 12 is consistent with our observations of a changing ion selectivity phenotype from Ca^2+^ to Na^+^ in exon 12a containing LCa_V_3 channels. We have analyzed the relative changes in Ca^2+^ and Na^+^ flux due to exon 12b and exon 12a in chimeras to engineer a Na^+^-selective T-type channel within a human Ca_V_3.2 T-type channel background and to engineer a Ca^2+^-selective T-type channel with the human extracellular L5_II_ loop onto a snail LCa_V_3 channel background (11). This is reflected in an increased contribution of the inward Na^+^ current estimated as the fold change in peak inward cation current size in physiological external Ca^2+^ concentration, when an equimolar quantity of external Na^+^ replaces weakly permeant monovalent ion, NMDG^+^. As we illustrate here and in previous publications (10, 11), the changing relative permeability of the Na^+^ flux compared to Ca^2+^ flux due to exon 12a can be estimated in bi-ionic reversal experiments as the degree of reversal potential change in response to Ca^2+^ influx to a competing efflux of different monovalent ions (Li^+^, Na^+^, K^+^, Cs^+^), in response to high concentrations of monovalent ions in internal solutions instead of external solutions.

### Bi-ionic reversal experiments do not adequately capture the magnitude of the changing ion permeabilities of molluscan Ca_V_3 channels, in the artificial conditions when monovalent ion flow is opposite to the inward Ca^2+^ current

The calculated relative change in the monovalent ion permeability estimated in bi-ionic reversal experiments are observed to be statistically significant (see Fig. 7) but do not appear to reflect the magnitude of the change when Na^+^ ions are observed as a competing inward current with external Ca^2+^ ions (*i.e.*, in experiments where external Na^+^ replaces external NMDG^+^, see Figs. 4 and 8). This is likely a consequence of the asymmetrical nature of the ion channel pore, where the consequences of neutralization of the aspartate in the outer pore by the lysine residue from the L5_II_ extracellular loop is experienced more dramatically when Ca^2+^ and Na^+^ ions are competing for binding sites as inward passing ions from the extracellular side of the pore. A blunting of the relative change in monovalent ion permeability in bi-ionic reversal experiments is understandable, when considering that monovalent ions like Na^+^ ions are not experiencing an equivalent competition with inward Ca^2+^ ions moving outward from the intracellular side of the ion channel pore. In bi-ionic reversal experiments, the outward flux of Na^+^ ions through the ion channel pore are approaching the outer pore aspartate residue, only after Na^+^ ions have transited through the negatively charged ion selectivity filter ring from the intracellular side of the pore. We believe that because a key determinant for the channel pore’s preference of Na^+^ and Ca^2+^ influx is on the external side of the ion channel pore, the aspartate in the outer pore can only effectively regulate the relative Ca^2+^ and Na^+^ permeability when Na^+^ and Ca^+^ ions are both competing as inward currents fluxing from the extracellular side. Given the asymmetrical nature of the regulator for Na^+^ and Ca^2+^ ion permeability on the extracellular side of the pore, the magnitude of the permeability changes specifically for the molluscan LCa_V_3 channels is not reflected in the calculated permeabilities from reversal potential values gathered using bi-ionic recording solutions.

We observe that the relative magnitude of changes to sodium permeation in molluscan Cav3-12a channels are more dramatically reflected in experiments which mimic more physiological conditions when Na^+^ is competing with Ca^2+^ as inward currents from the extracellular side. For example, traditional Ca^2+^-selective channels generate a “*U-shaped*” response curve in response to increasing doses of external calcium ions in the presence of external Na^+^ (10, 11). The U-shaped response to increasing Ca^2+^ is considered a reflection that Na^+^ ions are unable to compete for the cation binding sites as Ca^2+^ ions effectively repels the funneling of external Na^+^ through the Ca^2+^-selective pore in the presence of low 10 μM external Ca^2+^ concentrations and where Ca^2+^ influx dominates Na^+^ influx at physiological (mM) levels of external Ca^2+^ through calcium-selective channels (10, 11). The property of a high Na^+^ selectivity over Ca^2+^ in the molluscan LCa_V_3 channel pore containing exon 12a is reflected in the transformation of the “*U-shaped”* response curve in response to increasing external Ca^2+^ into a “*reverse S*” response curve, where low 10 μM external Ca^2+^ concentration is ineffective in the blocking of the Na^+^ influx, and rises in external Ca^2+^ to external physiological (mM) concentrations does not lead to greater Ca^2+^ passage through the Na^+^ selective T-type channel pore, but rather there is an observed increasing block of the T-type channel current because Ca^2+^ is not capable of permeating through the sodium-selective, LCav3-12a T-type channel pore as an inward current at physiological (mM) concentrations of Ca^2+^ (10, 11).

### All known mollusks and nematode Cav3 T-type channel transcripts possess predicted T-type sodium channels expressed with exon 12a

All available nematodes sequences surveyed in GenBank, including model organism, *C. elegans*, possess the lysine residue adjacent to the outer pore aspartate to generate a mostly Na^+^-passing T-type channel with their exon 12a like in mollusks (10, 11). All exon 12b sequences of nematodes possess a neutral residue (alanine, methionine, serine, and threonine) in identical position to the lysine residue to generate a mostly Ca^2+^ passing T-type channel (10, 11). The only notable structural difference between molluscan and nematode T-type channel with exon 12a and exon 12b is a different location of the cysteine that bridges from L5_II_ extracellular loop to the cysteine in the voltage sensor domain. The cysteine bridging the voltage sensor is within rather than outside the two pairs of intraturret cysteines in all protostome invertebrates outside of nematodes (Figs. 1 and 3).

### The differing turret cysteine bridging to the voltage-sensor domain in exon 12a generates a kinetically slower T-type sodium channel appropriate for the molluscan heart

The differing location of the cysteine tethered to the voltage sensor domain within extracellular loops of exon 12a and exon 12b might reflect the differing cellular conditions requiring kinetically fast or slow Cav3 T-type channels within protostome invertebrates. In the case of mollusks, they possess a traditionally fast, Na^+^-selective Na_V_1 channel, but this gene is limited in expression to the nervous system (10). A Na^+^-permeant T-type channel with exon 12a is the only isoform of T-type channel expressed in the molluscan heart where it serves as a surrogate Na^+^ current in the absence of expression of the singleton Na_V_1 channel in mollusks (10). Exon 12a imparts a dramatically slowing of its activation, inactivation, deactivation, and recovery from inactivation in LCa_V_3 T-type channels compared to exon 12b. This slowing of kinetics suits the requirements of a surrogate Na^+^ current in the snail heart, akin to a heart specific isoform in vertebrates, such as Na_V_1.5 (25), to establish a cardiac action potential with a prolonged repolarization phase required for refilling of the heart, with a built in refractoriness to prevent ectopic heart beats.

While the co-opting of a kinetically slower, Na^+^-permeant T-type channel serves the purposes of mollusks *in lieu* of expression of their Na_V_1 channel gene within molluscan hearts, *C. elegans* lack expression of any Na_V_2 or Na_V_1 sodium channel gene (26). Nematode Ca_V_3 T-type channel sequences generate precisely aligned extracellular turret structures in AlphaFold2 with identical positioning of the outer pore aspartate nullifying lysine residue with exon 12a and a precisely identical positioning for a neutral residue in exon 12b. But there is also a difference in nematode Ca_V_3 channels where the cysteine linkage to the voltage sensor from the L5_II_ extracellular loop is outside the one and two intraturret cysteine bridges for both exons 12a and exon 12b. The unique configuration of exon 12a and exon 12b within nematodes provide an optional sodium permeability for the nematode Ca_V_3 channel to meet expectations of action potential spike generation such as in the pharynx of *C. elegans*, where voltage-gated sodium currents have been observed (8, 9).

### Cnidarians appear to have evolved a separate Ca_V_3 channel gene to generate alternative sodium and calcium permeable T-type channels

Instead of custom-designing novel alternatively spliced exons coding for extracellular loops that serve to neutralize the aspartate in the ion selectivity filter (E) +1 position (ED) in Domain II, why don’t invertebrates just create an alternative Ca_V_3 gene, which just directly replaces the aspartate in the outer pore of Domain II to generate sodium permeable Ca_V_3 channels? This is what cnidarians apparently do (see Fig. 1*A*). Akin to molluscan LCav3, which generate sodium permeable Cav3 channels by neutralization of the outer pore aspartate with a custom-designed extracellular loop with exon 12a, cnidarians, such as stony coral *Porites australiensis* possess a separate PaCav3a gene, which contains a neutral asparagine (EN) in place of the aspartate (ED) in the outer pore of Domain II (Fig. 1*A*). The second cnidarian Cav3 gene, PaCa_V_3b, more resembles known calcium-selective Ca_V_1, Ca_V_2, and Ca_V_3 channels in eukaryotes, such as human Ca_V_3.3 with an outer pore with an aspartate in Domain II (ED) (Fig. 1*B*). The presence of a second Ca_V_3 channel gene within cnidarians is unique to the invertebrates, which normally possess a solitary Ca_V_3 gene in their genome. It is suggestive of a unique evolutionary pressure within the cnidarian lineage (mostly anthozoan cnidarians) to duplicate and create two separate Ca_V_3 T-type channels for the purposes to create alternative sodium and calcium permeable ion channel pores in a manner different form other invertebrate Ca_V_3 T-type channels, such as in mollusks and nematodes. The cnidarians are the simplest known eumetazoans with a sodium-selective Na_V_1 channel gene and the simplest known animals to possess a *true* nervous system. The cnidarians have a penchant for local adaptation, where the lysine residue in the ion selectivity filter ring of basal cnidarian Na_V_1 channels is uniquely positioned in Domain II (DKEA) instead of Domain III (DEKA) of the ion selectivity of other animal phyla (27). The lysine residue in the ion selectivity filter ring is a principal determinant for sodium permeation within Na_V_1 channels over the nonselective Na_V_2 channels (5), which are found in eukaryotic species outside mammals, with a DEEA ion selectivity filter ring (28). Notably, the Na_V_1 and Na_V_2 channels lack any residue in homologous position (K_) of the aspartate residue (ED) in the pore of Domain II. And it is the nullification of this aspartate residue charge featured in the outer pore in Domain II of calcium-selective channels that appears to be a key determinant for increasing sodium permeability within Ca_V_3 channels.

### An adjustable sodium and calcium permeability in invertebrate Ca_V_3 channels requires buttressing with additional intraturret disulfide bonds within L5_II_ and/or L6_IV_ extracellular loops

The different cnidarian Ca_V_3 genes, which vary in the presence or absence of an outer pore aspartate, also vary in the number of disulfide bonds within extracellular loops, with the cnidarian Ca_V_3a gene resembling the other invertebrate Cav3 channels of the invertebrates with two intraturret disulfide bonds in the L6_IV_ extracellular loop compared to one intraturret disulfide bond in the L6_IV_ extracellular loop of the cnidarian Ca_V_3b gene resembling the condition of the more calcium-selective, Ca_V_3 T-type channels of the vertebrates.

The importance of additional intraturret disulfide bonds in the L6_IV_ extracellular loop for generating sodium-permeable channels within the invertebrates was revealed in an exploration of the minimum determinants within molluscan LCav3-12a channels, required for generating high sodium permeation within human Cav3.2 channels which are normally more calcium than sodium permeant (11). Exon 12a from molluscan LCav3-12a conferred an increase in sodium permeability of human Ca_V_3.2 channels, but the magnitude of the sodium permeation only approached that of molluscan Cav3-12a channels when the L6_IV_ extracellular loop containing the nonvertebrate condition of two intraturret disulfide bonds also replaces the vertebrate L6_IV_ extracellular loop containing one intraturret disulfide bond (11).

The requirement of intraturret disulfide bonds within L5_II_ extracellular loops in generating sodium-permeable channels was also analyzed after discovering cysteine to alanine mutations in exon 12a and exon 12b of molluscan LCav3 channels (11). Disruption of the intraturret disulfide bridges in L5_II_ extracellular loop dramatically increased the Na^+^ permeability and weakened the capacity of external Ca^2+^ to block the Na^+^ current through both LCav3-12a(ΔCys) and LCav3-12b(ΔCys) mutants (11). Disruption of intraturret cysteine bonds also dramatically altered the ion pore’s accessibility to permeation and drug block to different divalent ions. Native LCav3 channels generate ∼1.4- and ∼1.3-fold larger peak barium (Ba^2+^) or strontium (Sr^2+^) currents, respectively, compared to equivalent Ca^2+^ currents (11). In ΔCys mutants of intraturret cysteines within L5_II_ extracellular loops, the relative peak currents of divalent ions within LCav3 channels reversed with peak Ba^2+^ or Sr^2+^ currents becoming a fraction (50% and 80%) of the much larger peak Ca^2+^ currents (11). Disruption of intraturret cysteine bonds also dramatically increased the potency of Zn^2+^ and Ni^2+^ block by ∼ 50- and ∼ 10-fold, respectively, including a change in drug blocking phenotype in the loss of the typical slowing of inactivation kinetics during Zn^2+^ block (11).

Our findings in analyzing the unique adaptations to generate sodium-permeable Ca_V_3 channels within invertebrates contribute to understanding mechanisms of Ca^2+^ and Na^+^ ion selectivity of eukaryotic Ca_V_ and Na_V_ channels (see Table 1). A disturbance in the highly electronegative ring composed of glutamates (E) and aspartates (D) of the ion selectivity filter with a lysine in Domain II or Domain III is what separates the Na^+^ permeable Na_V_1 channels found within the metazoans (5). Ca^2+^ permeability is defined by the near ubiquitous presence of an outer pore aspartate (D) in the ion selectivity filter +1 position in addition to the electronegative ring composed of glutamates (E) and aspartates (D) (29). Invertebrate Ca_V_3 channels can generate sodium permeable isoforms by neutralizing the outer pore aspartate (D) in Domain II to enable a variable sodium permeability, in the presence of extra intraturret disulfide bonds, especially within the short L5_II_ and L6_IV_ extracellular loops. The extra intraturret disulfide bonds are likely to serve as restraining straps, limiting the dynamic movements of the short extracellular loops, and stabilize the positioning of the ring of ion selectivity filter residues, the outer pore aspartate in Domain II neutralizing lysine residue found in exon 12a.

**Table 1:** Distribution of cysteine bridges in extracellular loops and key determinants for Na^+^ and Ca^2+^ permeation of Na_V_, Ca_V_, and NALCN channels

The additional cysteine bridges in CaV3 channels compared to NaV, CaV1/2, or NALCN channels are colorized in brown color instead of orange color. The negatively-charged amino acids (D/E) are colored in pink/red respectively. The positively-charged amino acid (K) are colored in blue.

^*a*^ NaV2 and NaV1 channels, including vertebrate NaV1.x (where x=1,2,3,4,6,7) can possess up to three additional S-S: L5II, P2II to L6II, L5II to NaVβ2 or NaVβ4

## Experimental procedures

### Reagents and tools table

**Table:** 

Reagent or resource	Source	Identifier
Chemicals, peptides, and recombinant proteins		
Dulbecco’s modified Eagle’s medium	Millipore Sigma-Aldrich	Cat # D5796
Sodium pyruvate	Millipore Sigma-Aldrich	Cat # S8636
Fetal bovine serum	Wisent Bioproducts	Cat # 080–910
Penicillin (10,000 U/ml) streptomycin (10 mg/ml)	Millipore Sigma-Aldrich	Cat # P4333
Trypsin-EDTA solution	Millipore Sigma-Aldrich	Cat # T4049
Poly-L-lysine solution	Millipore Sigma-Aldrich	Cat # P4707
Hepes	Millipore Sigma-Aldrich	Cat # 391338
Experimental models: cell lines		
HEK-293T	ATCC	Cat # CRL-3216
Recombinant DNA		
LCav3-12a (Cav3 T-type channel from *Lymnaea stagnalis* with configuration + exon 8b, + exon 12a, - exon 25C)	Senatore *et al.* 2010	RRID: Addgene ID#: 185536
LCav3-12b (Cav3 T-type channel from *Lymnaea stagnalis* with configuration + exon 8b, + exon 12b, - exon 25C)	Senatore *et al.* 2014	RRID: Addgene ID#: 185535
Software and algorithms		
pClamp10	Molecular Devices	https://www.moleculardevices.com/products/axon-patch-clamp-system/acquisition-and-analysis-software/pclamp-software-suite
Origin-Pro 2017	Originlab	https://www.originlab.com/origin
AlphaFold v2.0	Google	https://github.com/deepmind/alphafold
PYMOL 2.5	Schrödinger	https://pymol.org/2/
ChimeraX	Resource for Biocomputing, Visualization, and Informatics at UCSF	https://www.rbvi.ucsf.edu/chimerax/

Further information and requests for resources and reagents should be directed to and will be fulfilled by the Lead Contact, J. David Spafford (spafford@uwaterloo.ca).

### Data availability statement

Addgene catalog numbers are given in Key Resources table.

## Experimental model details

### HEK-293T cell line

HEK-293T cell lines (M. Calos, Stanford University) were plated onto cell culture flasks and coverslips coated with poly-L-lysine and cultured in a 5% CO2 incubator at 37 °C. HEK-293T cells were cultured in Dulbecco's modified Eagle's medium (Millipore Sigma-Aldrich) with 10% fetal bovine serum (Millipore Sigma-Aldrich) and supplemented with 0.5% (v/v) penicillin-streptomycin solution (Millipore Sigma-Aldrich).

## Method details

### Molecular biology

The mostly Ca^2+^ passing isoform of the invertebrate LCa_V_3 T-type channel (GenBank Accession #: AAO83843) isolated from pond snail, *L. stagnalis*, was expressed and characterized in a configuration contained exon 12b, as well as optional exon 8b spanning the I–II linker but lacking exon 25c of the III–IV linker (30). Novel exon 12a isoform (+8b, −25C) deposited as GenBank Accession # AFN89594, is compared with exon 12b isoform (+8b, −25C), which is the configuration of the three exons that is more commonly expressed in the snail brain than in the snail heart where there is exclusive expression of the mostly Na^+^ current passing Ca_V_3 T-type channel with exon 12a (10). Human Cav3.1 and Cav3.2 channels were a gift provided by Edward Perez-Ryes, Department of Pharmacology, University of Virginia.

Site-directed mutations within exon 12 of full-length Cav3 channels (LCav3-12a_K1067A, LCav3-12a_D1087N, LCav3-12b_A1089K) were generated by swapping synthetic gene fragments containing the site-specific mutations (ordered from BioBasic Canada) spanning novel silent restriction sites AvrII and Eco47III (AfeI) introduced at positions: 3338 to 3567 spanning the coding sequence for exon 12a (39 amino acids) and exon 12b (50 amino acids). Synthetic DNA (ordered from BioBasic Canada) spanning the AvrII and Eco47III restriction sites were inserted into a subclone in pGEMT vector at unique BglII and SalI restriction sites (positions: 1391–4521). Successfully ligated subclones containing the site-directed mutations were reintroduced into the full-length LCav3 cDNA cloned in pIRES2-EGFP at the BglII and SalI restriction sites. LCav3-12a_K1067A, LCav3-12a_D1087N, and LCav3-12b_A1089K contained a unique BspEI, PvuI, and KpnI silent restriction sites, respectively, for rapid validation of individual cloned plasmid stocks for their unique L5_II_ extracellular loop identity in LCa_V_3. The method used to create site-directed mutation of Cav3.2 at D975N was described previously (11).

### Molecular modeling

AlphaFold2 (31) was used to generate molecular models of the molluscan Cav3 T-type channel from *L. stagnalis* containing exon 12b (Genbank Acc. # AAO83843) or containing exon 12a (Genbank Acc. # JX292155) and models of nematode Cav3 T-type channel from *C. elegans* containing exon 12b (Genbank Acc. # AAP79881.1) or containing exon 12a (Genbank Acc. # AAP84337.1). Structural models were overlaid and curated in ChimeraX (Resource for Biocomputing, Visualization, and Informatics at UCSF) or PyMOL 2.5 (Schrödinger).

### Cell line transfection

LCav3 clones including mutated derivatives were expressed within bi-cistronic, mammalian expression, pIRES2-EGFP vector, which generates green fluorescence upon mercury lamp excitation for identifying positively transfected HEK-293T cells. Human Cav3.1 and Cav3.2 were cotransfected with EGFP reporter gene containing plasmid, pEGFP-C1 (Takara Bio).

Six micrograms of Cav3 channel containing plasmids were heterologously expressed by calcium-phosphate transfection into human embryonic kidney cell line (HEK293T, M. Calos, Stanford University) at 40 to 50% confluency. After overnight transfection, the cells were washed three times with culture media and incubated at 28 °C in a humidified, 5% CO_2_ chamber for 3 to 5 days. Cells were replated in 60 mm (diameter) sterile Petri dishes containing eight poly-lysine coated glass coverslips (Circles No. 1–0.13 to 0.17 mm thick; Size: 12 mm) (ThermoFisher Scientific) and allowed to recover at 37 °C for 4 hours and then left at 28 °C for at least 3 days before patching. Complete details of our methods for optimized transfection and recording of ion channels is available as a video journal entry at: http://www.jove.com/index/Details.stp?ID=2314 (32).

### Electrophysiology

Whole cell electrophysiology recordings were obtained using an Axopatch 200B or Multiclamp 700B amplifiers (Molecular Devices), sampled through a Digidata 1440a A/D converter (Molecular Devices) to a PC computer. Patch pipettes for recording transfected HEK-293T cells had pipette resistances of 2.5 to 5 MΩ and with typical access resistance maintained after breakthrough between 2.5 MΩ and 6 MΩ (HEK-293 T cells). Voltage clamp protocols included a holding and intersweep potential of −110 mV, which is a potential that Cav3 calcium channels are not subject to inactivation. The intersweep interval was set to at least 30 seconds for the molluscan LCav3 channels, which is the minimum time for their complete recovery from inactivation. All voltage steps were carried out for 300 ms that enabled a full display of the decay of inactivation kinetics, at all voltage steps from −80 mV to +80 mV. The peak ionic currents of molluscan Cav3 channels are left-shifted by ∼10 mV compared to human Cav3.2 channels, so voltage steps to peak current were displayed at voltage steps to −40 mV for molluscan Cav3 channels and −30 mV for human Cav3.2 channels in Fig. 4 or Fig. 8.

Series resistance was compensated to 70% (prediction and correction; 10-μs time lag). Offline leak subtraction was carried out using the ClampFit 10.2 software (Molecular Devices). For all recordings, offline leak subtraction was carried out, and data were filtered using a 500 Hz Gaussian filter in Clampfit 10.2. A ValveLink 8.2 gravity flow Teflon perfusion system (AutoMate Scientific) was utilized to compare ionic current sizes generated by external monovalent or divalent ions.

Calcium currents through Cav3 T-type channels were recorded in the presence in external calcium containing extracellular recording solution consisting of (in mM): 2 CaCl_2_, 135 NMDG^+^, 25 TEA (tetraethylammonium), 10 Hepes (4-(2-hydroxyethyl)-1-piperazineethanesulfonic acid), at a pH of 7.4 titered with TEA-OH. Internal recording solution consisted of (in mM): 110 CsCl, 10 EGTA, 3 Mg-ATP, 0.3 Tris-GTP, and 10 Hepes, at a pH of 7.2 titered with CsOH. The evaluation of the relative sodium conductance through LCav3 T-type channels were evaluated in the presence of the external calcium-containing extracellular recording solution described above, where 135 mM Na^+^ replaced 135 mM NMDG^+^.

The relative permeability of PCa/Px was derived from reversal potentials extrapolated from the linear conductances of current-voltage relationships spanning the reversal potential using the following bi-ionic solutions: extracellular recording solution consisting of (in mM): 4 CaCl_2,_ 155 TEA (tetraethylammonium), 10 Hepes (4-(2-hydroxyethyl)-1-piperazineethanesulfonic acid), at a pH of 7.4 titered with TEA-OH. Internal recording solution consisted of (in mM): 100 CsCl or NaCl or KCl or LiCl, 10 EGTA, 10 Hepes, and pH of 7.2, titered with XOH, where X = Cs, Na, K, Li.

The relative permeability of PCa/Px was calculated by the following bi-ionic equation provided on the bottom of page 34 of Fatt and Ginsborg (1958) (23):

$$\frac{PCa}{Px}=\frac{{\left[x\right]}_{internal}}{4{\left[Ca\right]}_{external}}\exp\left(\frac{E_{rev}F}{RT}\right)\left\{\exp\left(E_{rev}F/RT\right)+1\right\}$$

where x is a monovalent ion (Li^+^, Na^+^, K^+^, Cs^+^), F = Faraday constant, R = ideal gas constant, T = temperature (°K) and E_rev_ = reversal potential.

## Quantification and statistical analyses

Electrophysiology data gathered using pClamp10 (Molecular Devices) was imported into Origin-Pro 2017 (OriginLab Corporation) for statistical analyses. Kinetics of activation, inactivation, and deactivation were determined by fitting monoexponential functions over the growing or decaying phases of each current trace. Electrophysiology data are shown as mean ± SEM, where “n” refers to number of cells, provided in the figure legends, together with details of statistical tests used. Statistical significance between two groups was assessed by Student’s *t* test, as stated. One-way ANOVA and the stated post hoc analysis were used for comparison of means between three or more groups. At least three independent transfections of HEK-293T cells were used to gather the data collected in Figs. 4 and Figure 6, Figure 7, Figure 8.

## Conflict of interest

The authors declare that they have no conflicts of interest with the contents of this article.
